# Intentional Large-Volume Diazinon Ingestion Complicated by Seizure and Acute Respiratory Failure: A Case Report

**DOI:** 10.7759/cureus.111774

**Published:** 2026-06-29

**Authors:** Khuloud H Alnuaimi, Salama Alharmoodi, Reem Almerri, Mouza Alneyadi, Aysha Al Harthi

**Affiliations:** 1 Emergency Department, Sheikh Tahnoon Bin Mohammed Medical City (STMC), Al-Ain, ARE

**Keywords:** atropine, cholinergic toxicity, diazinon, organophosphate poisoning, pralidoxime, respiratory failure, seizure

## Abstract

Organophosphate poisoning is a major cause of acute cholinergic toxicity and can progress rapidly to respiratory failure and death. In the emergency setting, diagnosis is often clinical, and treatment should not be delayed while awaiting confirmation of the specific compound. We report a case of a 30-year-old male patient who presented after intentional ingestion of a large reported volume of diazinon-containing pesticide. Within approximately 30 minutes of ingestion, he developed acute cholinergic manifestations, including bradypnea, bradycardia, hypotension, hypersalivation, vomiting, diarrhea, and depressed level of consciousness, complicated by a generalized tonic-clonic seizure. The seizure was treated with diazepam, and atropine was initiated before transfer to the emergency department. On arrival, the patient was in severe respiratory distress with profuse secretions and required urgent endotracheal intubation and mechanical ventilation. Empiric treatment for suspected organophosphate poisoning was continued with atropine and pralidoxime, alongside supportive care. Collateral history later identified the ingested product as Diacidol 600 Emulsifiable Concentrate, containing diazinon 600 g/L, equivalent to 60% weight/volume. Plasma butyrylcholinesterase, red blood cell acetylcholinesterase, and toxicological confirmation testing were not available at our institution at the time of evaluation, which represents a limitation of this report. The patient was admitted to the intensive care unit and managed with atropine and pralidoxime infusions, ventilatory support, and close monitoring for delayed neuromuscular complications. His cholinergic features resolved, and he was successfully extubated within 24 hours. Antidotal therapy was discontinued after sustained clinical improvement, and he remained stable during subsequent ward observation. This case emphasizes that severe organophosphate poisoning remains a clinical diagnosis during initial resuscitation. Early airway protection, titrated atropinization, pralidoxime therapy, benzodiazepines for seizures, and observation for delayed complications are central to favorable outcomes.

## Introduction

Organophosphate poisoning remains a significant global health problem and a common cause of acute toxic exposure, particularly in regions where agricultural pesticides are widely available. Organophosphate compounds inhibit acetylcholinesterase, resulting in the accumulation of acetylcholine at muscarinic, nicotinic, and central nervous system receptors. The resulting cholinergic toxidrome may include hypersalivation, bronchorrhea, bronchospasm, bradycardia, hypotension, gastrointestinal hyperactivity, miosis, fasciculations, altered mental status, seizures, respiratory failure, and death [1].

Early diagnosis is primarily clinical and may be challenging when the exact agent or exposure history is initially unknown. In unstable patients, management should not be delayed while awaiting laboratory confirmation or identification of the specific compound. Prompt decontamination, airway support, atropine, pralidoxime, seizure control, and critical care monitoring are essential components of treatment [2-4].

Diazinon is an organophosphate pesticide that produces toxicity through acetylcholinesterase inhibition after metabolic activation. Severe ingestion may result in a rapid cholinergic crisis, respiratory failure, cardiovascular instability, and central nervous system manifestations. Although altered consciousness and coma are well-recognized neurological features of severe organophosphate poisoning, seizures are less commonly reported following organophosphate pesticide ingestion than after nerve agent exposure and require prompt benzodiazepine treatment [1].

We report a case of intentional large-volume diazinon ingestion complicated by an early generalized tonic-clonic seizure and acute respiratory failure. This case highlights the importance of early clinical recognition, empiric antidotal therapy, airway protection, and monitoring for delayed complications.

## Case presentation

A 30-year-old male patient with an unknown past medical history was brought to the emergency department by emergency medical services as a referral from a primary healthcare facility after intentional pesticide ingestion in a suicide attempt. His past medical history, regular medications, psychiatric diagnosis, prior suicide attempts, and triggering event were not available from the initial record. He initially presented to the primary clinic approximately 30 minutes after ingestion. At that time, he had features consistent with acute cholinergic toxicity, including bradypnea, bradycardia, hypotension, hypersalivation, recurrent vomiting, diarrhea, and depressed level of consciousness, with a Glasgow Coma Scale score of 10/15. He was transferred to the emergency department shortly thereafter, within approximately one hour of the initial primary healthcare presentation.

During the initial evaluation at the primary healthcare facility, he developed a witnessed generalized tonic-clonic seizure, which was terminated with 10 mg of diazepam. Initial treatment before transfer included atropine, with a cumulative dose of 7 mg, and metoclopramide.

Before the patient’s arrival at the emergency department, the receiving team prepared personal protective equipment because of suspected pesticide exposure and the potential risk of secondary contamination, rather than as a routine measure for all suicide attempts. Respiratory therapy and anesthesiology teams were alerted because of the anticipated need for airway intervention. On arrival, the patient underwent decontamination before transfer to the resuscitation area.

In the emergency department, he was confused and in severe respiratory distress. Vital signs showed a heart rate of 130 beats/min, respiratory rate of 35 breaths/min, blood pressure of 129/83 mmHg, and oxygen saturation of 97% on 15 L/min oxygen via a non-rebreather mask. The change from initial bradycardia to tachycardia was considered likely related to prior atropine administration and evolving physiological stress. Physical examination showed profuse respiratory secretions and bilateral crackles on chest auscultation. Cardiovascular examination revealed normal heart sounds, palpable peripheral pulses, and warm extremities. The abdomen was soft and non-tender, with no other major abnormalities identified.

Because of severe bronchorrhea, tachypnea, depressed consciousness after seizure, and high aspiration risk, the patient underwent urgent endotracheal intubation for airway protection and management of acute respiratory failure. In our institution, immediate airway management in the resuscitation area is performed by the emergency department team when clinically indicated, with anesthesiology support available if required. Intubation was performed by the emergency team using fentanyl, midazolam, and rocuronium. Post-intubation sedation was maintained with fentanyl and propofol. He received intravenous fluids, additional atropine reaching a total cumulative dose of 12 mg, and pralidoxime 2 g intravenously as initial antidotal therapy for suspected organophosphate poisoning.

Following stabilization, collateral history was obtained from the patient’s colleagues and law enforcement. The ingested substance was identified as Diacidol 600 Emulsifiable Concentrate, a pesticide containing diazinon 600 g/L, equivalent to 60% weight/volume. The reported ingested volume was approximately 700 mL. This would correspond to a very large potential exposure, up to approximately 420 g of diazinon if the reported volume and concentration were accurate. However, the actual absorbed dose could not be confirmed. The estimate was based on collateral history, and the bottle residual volume was not reliably documented. Early vomiting and incomplete gastrointestinal absorption may also have reduced systemic exposure.

Laboratory investigations were performed to assess acid-base status, metabolic derangements, co-ingestion, and alternative or contributing causes of seizure. Plasma butyrylcholinesterase, red blood cell acetylcholinesterase, methanol level, and comprehensive toxicological confirmation testing were not available at our institution at the time of evaluation. Therefore, the diagnosis was based on the compatible cholinergic toxidrome, temporal relationship to ingestion, collateral identification of the product, and clinical response to organophosphate-directed therapy.

Key laboratory findings are summarized in Table 1. 

**Table 1:** Laboratory findings on presentation NT-proBNP: N-terminal pro-B-type natriuretic peptide; ECG: electrocardiogram; pCO_2_: partial pressure of carbon dioxide

Parameter	Result	Reference range
pH, venous blood gas	7.28	7.35-7.45
pCO₂	35 mmHg	35-45 mmHg
Bicarbonate	16 mmol/L	22-28 mmol/L
Base excess	-9	-2 to +2
Lactate	6 mmol/L	0.5-2.0 mmol/L
Sodium	144 mmol/L	135-145 mmol/L
Potassium	3.2 mmol/L	3.5-5.0 mmol/L
Chloride	111 mmol/L	98-107 mmol/L
Carbon dioxide	14 mmol/L	22-28 mmol/L
Anion gap	19 mmol/L	8-16 mmol/L
Creatinine	79.7 µmol/L	-
Urea	3.5 mmol/L	-
Corrected calcium	2.31 mmol/L	2.10-2.60 mmol/L
Glucose	11.8 mmol/L	3.9-7.8 mmol/L
Serum ethanol	24.5 mmol/L, approximately 113 mg/dL	<1 mmol/L
White blood cell count	22.7 ×10⁹/L	4.0-11.0 ×10⁹/L
Hemoglobin	159 g/L	130-170 g/L
Platelets	265 ×10⁹/L	150-400 ×10⁹/L
Total creatine kinase	131 U/L	-
Troponin-I	24.1 ng/L	-
NT-proBNP	22 ng/L	-
Osmolality	333 mOsm/kg	275-295 mOsm/kg
Plasma butyrylcholinesterase	Not performed	-
Red blood cell acetylcholinesterase	Not performed	-
Methanol level	Not performed	-
Toxicological confirmation	Not performed	-
ECG	Sinus tachycardia	-

Venous blood gas analysis demonstrated metabolic acidosis with elevated lactate. Initial chemistry showed low carbon dioxide, elevated anion gap, mild hypokalemia, mild hyperglycemia, and preserved renal function. The lactate elevation was interpreted as multifactorial, likely related to the witnessed generalized seizure, respiratory distress, and systemic stress from severe poisoning. Serum ethanol was elevated at 24.5 mmol/L, approximately 113 mg/dL, and measured osmolality was elevated at 333 mOsm/kg, which may have contributed to depressed consciousness and aspiration risk. Complete blood count showed leukocytosis, likely reflecting physiological stress. Creatine kinase was not significantly elevated, and initial troponin-I was mildly elevated with a small rise on repeat testing, without documented ischemic electrocardiogram (ECG) changes.

Serial blood gas monitoring demonstrated improvement in acid-base status after resuscitation, airway control, and antidotal therapy. The initial metabolic acidosis improved over time, with pH increasing from 7.28 on presentation to 7.38 on subsequent testing, while bicarbonate improved from 16 mmol/L to approximately 20-24 mmol/L.

Co-ingestion was considered because of the elevated serum ethanol level and metabolic acidosis. Although methanol testing was not available during the acute evaluation, the patient’s presentation was dominated by a typical cholinergic toxidrome, including hypersalivation, bronchorrhea, bradypnea, bradycardia before atropine administration, gastrointestinal hyperactivity, and depressed consciousness, with a close temporal relationship to reported pesticide ingestion. Collateral history later identified the ingested product as diazinon-containing Diacidol 600 Emulsifiable Concentrate. There were no documented visual symptoms, and the patient improved rapidly with atropine, pralidoxime, ventilatory support, and supportive care. Therefore, organophosphate poisoning was considered the primary diagnosis, although unconfirmed co-ingestion could not be completely excluded.

Chest radiography confirmed appropriate endotracheal tube position and showed no focal consolidation, pulmonary edema, or radiographic evidence of aspiration changes (Figure 1). Non-contrast computed tomography of the head showed no acute intracranial hemorrhage, mass effect, midline shift, or other structural abnormality (Figure 2).

**Figure 1:**
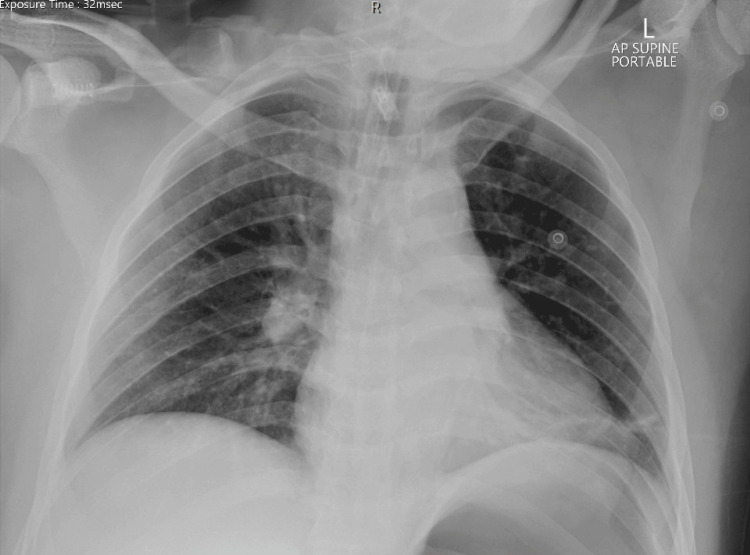
Chest radiograph showing appropriate endotracheal tube positioning, with no evidence of focal consolidation, pulmonary edema, or aspiration changes.

**Figure 2:**
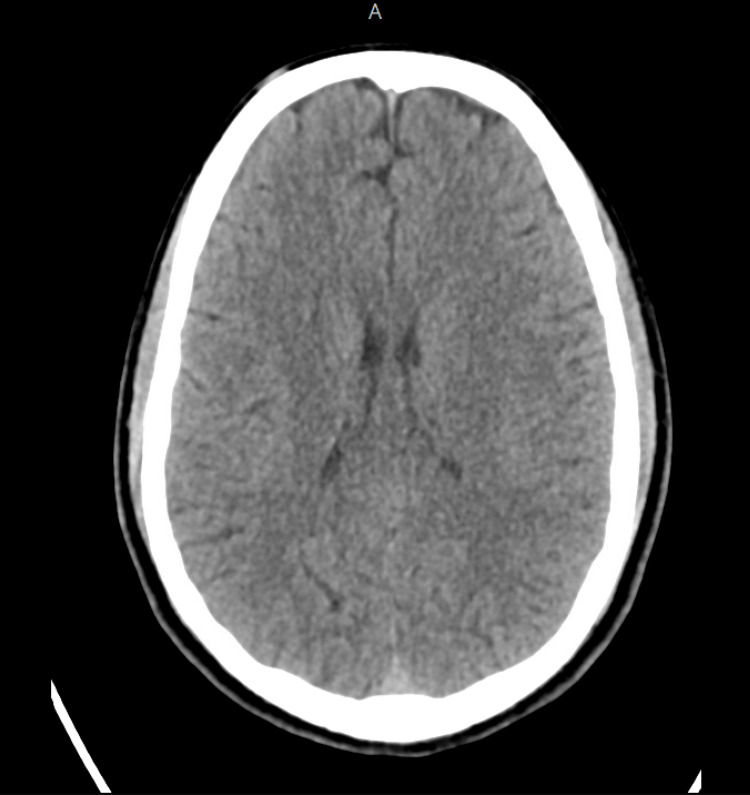
Non-contrast computed tomography of the head demonstrating no evidence of acute intracranial hemorrhage, mass effect, midline shift, or other structural abnormality.

The poison control center and intensive care unit teams were consulted promptly. The patient was admitted to the intensive care unit for mechanical ventilation, continuous atropine infusion, pralidoxime infusion, and close monitoring. During mechanical ventilation, he was managed using assist-control/volume-control mode with a fraction of inspired oxygen of 40%. Cardiovascularly, he remained hemodynamically stable, with no signs of shock or tissue hypoperfusion and no documented vasopressor requirement. Renal function remained preserved, with urine output of approximately 60-70 mL/hour; the documented fluid balance was positive by 2.4 L over the preceding 24 hours. His abdomen remained soft, and he was kept nil per os during the early critical care period. Supportive ICU care included peripheral intravenous access, proton pump inhibitor therapy for gastrointestinal prophylaxis, low molecular weight heparin for venous thromboembolism prophylaxis, and ceftriaxone during admission.

Atropine therapy was titrated to clinical response, particularly reduction of bronchorrhea, improvement in respiratory secretions, stabilization of hemodynamics, and improvement in chest auscultation. The exact atropine infusion rate was not documented in the available record. Pralidoxime was administered as a loading dose of 30 mg/kg over 15-30 minutes, followed by a maintenance infusion of 8-10 mg/kg/hour.

The patient improved progressively, with a reduction in secretions and stabilization of respiratory status. He was extubated within 24 hours of admission while continuing atropine and pralidoxime infusions. After extubation, he maintained oxygen saturation above 94% on 2 L/min oxygen via nasal cannula with comfortable breathing, remained hemodynamically stable, and was awake and alert. The atropine infusion was reduced gradually. Antidotal infusions were discontinued on the third day of admission after sustained resolution of cholinergic manifestations without recurrence of bronchorrhea, bradycardia, hypotension, or respiratory distress.

During hospitalization, he was monitored for delayed complications of organophosphate poisoning, including intermediate syndrome, proximal muscle weakness, neck flexor weakness, cranial nerve involvement, recurrent respiratory insufficiency, and need for re-intubation. None of these delayed neuromuscular or respiratory complications was documented during the inpatient stay. Because of the elevated ethanol level at presentation, he was also monitored for possible alcohol withdrawal during hospitalization.

Following medical stabilization, the patient was assessed by the psychiatric team and transferred to a monitored medical ward with level 2 observation according to psychiatric recommendations. He remained clinically stable and was discharged in stable condition in police custody after a medical and psychiatric assessment. No recurrent suicide attempt or readmission was documented in the available hospital record during the reviewed period. However, long-term psychiatric follow-up data were not available, which represents a limitation of this report.

The clinical course and key management milestones are summarized in Table 2.

**Table 2:** Timeline of clinical course ED: emergency department; IV: intravenous; ICU: intensive care unit; EC: Emulsifiable Concentrate; FiO_2_: fraction of inspired oxygen

Time	Event
0 min	Intentional ingestion of a reported approximately 700 mL diazinon-containing pesticide
Approximately 30 min	Presentation to primary healthcare facility with cholinergic symptoms, including bradypnea, bradycardia, hypotension, hypersalivation, vomiting, diarrhea, depressed consciousness, and witnessed generalized tonic-clonic seizure treated with diazepam 10 mg
Pre-transfer	Atropine administered; cumulative dose 7 mg
Within approximately one hour of primary healthcare presentation	Transfer by emergency medical services to the emergency department
ED arrival	Confusion, severe respiratory distress, profuse secretions, tachypnea, and bilateral crackles
Early ED course	Decontamination, airway preparation, urgent endotracheal intubation, and mechanical ventilation
ED treatment	Additional atropine, reaching a cumulative total of 12 mg; pralidoxime 2 g IV; supportive care
After stabilization	Collateral history identified Diacidol 600 EC containing diazinon 600 g/L
ICU admission	Mechanical ventilation using assist-control/volume-control mode with FiO₂ 40%; continuous atropine infusion titrated to clinical response and pralidoxime infusion started
Within 24 hours	Clinical improvement and successful extubation while continuing atropine and pralidoxime infusions
After extubation	Maintained oxygen saturation above 94% on 2 L/min oxygen via nasal cannula with comfortable breathing; remained hemodynamically stable, awake, and alert
Day 3	Antidotal infusions discontinued after sustained resolution of cholinergic features
Ward course	Psychiatric assessment, level 2 observation, and monitoring for alcohol withdrawal and intermediate syndrome
Discharge	Discharged clinically stable in police custody after medical stabilization and psychiatric assessment

## Discussion

This case highlights several important aspects of severe organophosphate poisoning, including early clinical recognition, prompt airway protection, timely antidotal therapy, and monitoring for delayed complications. Organophosphate compounds inhibit acetylcholinesterase, resulting in the accumulation of acetylcholine at muscarinic, nicotinic, and central nervous system receptors. The resulting cholinergic crisis can produce life-threatening manifestations, including bronchorrhea, bronchospasm, bradycardia, hypotension, altered mental status, seizures, respiratory failure, and death [1].

A notable feature in this case was the early occurrence of a generalized tonic-clonic seizure. Seizures are well recognized after nerve agent exposure but are less commonly reported following organophosphate pesticide ingestion. When present, they may indicate significant central nervous system toxicity and should prompt immediate treatment [1]. In this patient, the seizure occurred shortly after ingestion and was successfully terminated with diazepam. Early benzodiazepine administration is important because delayed treatment may increase the risk of prolonged or refractory seizures and neurological injury [2-4]. However, seizure etiology in acute poisoning may be multifactorial. In this case, organophosphate-related central cholinergic toxicity was considered the most likely contributor because the seizure occurred in close temporal association with pesticide ingestion and was accompanied by a clear cholinergic toxidrome, including hypersalivation, bronchorrhea, bradypnea, bradycardia before atropine administration, gastrointestinal symptoms, and depressed consciousness. Other possible contributors, including hypoxia, metabolic acidosis, ethanol intoxication, electrolyte disturbance, hypoglycemia or hyperglycemia, structural intracranial pathology, and mixed ingestion, were also considered.

The elevated serum ethanol level was clinically relevant because it may have contributed to depressed consciousness and aspiration risk. Alcohol withdrawal was considered unlikely as the cause of the initial seizure because the seizure occurred early after ingestion in the setting of acute ethanol exposure rather than after a period of abstinence. However, the patient was monitored for possible alcohol withdrawal during hospitalization. Methanol testing and comprehensive toxicological confirmation were unavailable during the acute evaluation, a limitation of this report. Nevertheless, there were no documented visual symptoms, computed tomography of the head showed no acute structural abnormality, glucose and electrolyte abnormalities were not sufficient to explain the seizure, and the patient improved rapidly with organophosphate-directed therapy. Therefore, organophosphate poisoning was considered the primary diagnosis, although unconfirmed co-ingestion could not be completely excluded.

Alcohol co-ingestion is considered a significant risk factor and is commonly reported in intentional pesticide poisoning. Further, it may complicate initial assessment by clouding the clinical picture. It has been associated with worse outcomes in pesticide poisoning, including increased mortality and higher rates of intubation. This may be related to behavioral factors, such as large ingestion volumes, and potential pharmacokinetic interactions that prolong toxin elimination [5,6]. 

Management of severe organophosphate poisoning is time-sensitive and requires decontamination, airway support, atropine, oxime therapy, seizure control, and intensive care monitoring. Respiratory failure is a major cause of morbidity and mortality and may result from excessive secretions, bronchospasm, aspiration, central respiratory depression, or neuromuscular weakness [1]. In this case, urgent endotracheal intubation was appropriate because of severe bronchorrhea, tachypnea, depressed consciousness after seizure, and high aspiration risk. Early airway protection allowed safe ventilation while antidotal therapy was continued. The preparation of personal protective equipment and decontamination was related to suspected pesticide exposure and the potential risk of secondary contamination to healthcare staff, rather than being a routine measure for all suicide attempts.

Atropine remains the mainstay of treatment for muscarinic manifestations of organophosphate toxicity. It should be titrated rapidly to clinical endpoints, including drying of bronchial secretions, improvement in oxygenation and ventilation, stabilization of heart rate and blood pressure, and improvement in chest auscultation, rather than to a fixed cumulative dose [7]. In this patient, atropine was initiated before transfer and continued in the emergency department and intensive care unit, with subsequent continuous infusion. Pralidoxime was also administered early. Oxime therapy may restore acetylcholinesterase activity if given before irreversible enzyme aging occurs, and continuous infusion is commonly used in severe poisoning to maintain therapeutic levels [8]. The patient’s rapid reduction in secretions, stabilization of respiratory status, and successful extubation within 24 hours underscore the importance of early combined airway management, atropine, pralidoxime, and supportive management.

Although the patient demonstrated rapid clinical improvement, delayed complications remained an important concern. Intermediate syndrome is a recognized delayed complication of organophosphate poisoning that usually occurs after resolution of the initial cholinergic crisis, commonly within 24-96 hours after exposure [9]. It is characterized by proximal muscle weakness, neck flexor weakness, cranial nerve involvement, and recurrent respiratory insufficiency [9]. Therefore, continued monitoring is essential even after apparent initial recovery. In this case, the patient was monitored for intermediate syndrome, recurrent respiratory insufficiency, and the need for re-intubation. None of these delayed neuromuscular or respiratory complications was documented during the inpatient stay.

Several laboratory abnormalities in this case supported the severity of the acute presentation but should be interpreted in the clinical context. The patient had metabolic acidosis with elevated lactate, mild hypokalemia, mild hyperglycemia, leukocytosis, and elevated serum osmolality. The lactate elevation was likely multifactorial, related to the witnessed generalized seizure, respiratory distress, and systemic stress from severe poisoning. Hypokalemia and hyperglycemia have been described in severe organophosphate poisoning, but in this patient, they were mild and were not considered sufficient to explain the seizure. These findings were therefore interpreted as supportive markers of acute physiological stress rather than definitive predictors of outcome [10,11]. Despite the severity of presentation, the absence of persistent shock and the early initiation of appropriate therapy likely contributed to the favorable short-term outcome.

Long-term neurological and psychiatric outcomes are important considerations after severe organophosphate poisoning. Acute central nervous system toxicity may be followed by cognitive, neuropsychiatric, or seizure-related complications in some patients. However, the risk varies according to exposure severity, treatment delay, and individual factors [12,13]. Given the early generalized seizure in this case, neurological follow-up would be reasonable. In addition, because the ingestion occurred in the context of a suicide attempt, psychiatric assessment and safety planning were essential. The patient was assessed by psychiatry during hospitalization and transferred to a monitored ward with level 2 observation before discharge. No recurrent suicide attempt or readmission was documented in the available hospital record during the reviewed period. However, long-term psychiatric follow-up data were not available to the authors, which represents a limitation of this report.

This case reinforces that severe organophosphate poisoning remains a clinical diagnosis during initial resuscitation, particularly when cholinesterase assays or toxicological confirmation are unavailable. The presence of a seizure should prompt benzodiazepine treatment but should not delay empiric organophosphate-directed therapy when the clinical features are consistent with cholinergic toxicity. Early decontamination, airway protection, titrated atropinization, pralidoxime therapy, and continued monitoring for delayed respiratory or neuromuscular complications are central to improving outcomes.

## Conclusions

Severe organophosphate poisoning remains a clinical diagnosis during initial emergency resuscitation, particularly when cholinesterase assays or toxicological confirmation are unavailable. This case illustrates the importance of early recognition of the cholinergic toxidrome, timely decontamination, airway protection in the presence of bronchorrhea and depressed consciousness, and prompt empiric antidotal therapy. Seizures, although less commonly reported following organophosphate pesticide ingestion than after nerve agent exposure, should be treated promptly with benzodiazepines while atropine and pralidoxime therapy are continued. Continued monitoring after initial improvement is essential to detect delayed respiratory or neuromuscular complications, including intermediate syndrome.
